# Sphingosine 1-Phosphate Signaling as a Target in Hepatic Fibrosis Therapy

**DOI:** 10.3389/fphar.2017.00579

**Published:** 2017-08-25

**Authors:** Bárbara González-Fernández, Diana I. Sánchez, Javier González-Gallego, María J. Tuñón

**Affiliations:** ^1^Institute of Biomedicine, University of León León, Spain; ^2^Centro de Investigación Biomédica en Red de Enfermedades Hepáticas y Digestivas (CIBERehd) León, Spain

**Keywords:** sphingosine 1-phosphate, sphingosine kinases, S1P receptors, hepatic stellate cells, fibrogenesis, liver fibrosis, target therapy, inhibitors

## Abstract

Liver fibrosis is an excess production of extracellular matrix proteins as a result of chronic liver disease which leads to cell death and organ dysfunction. The key cells involved in fibrogenesis are resident hepatic stellate cells (HSCs) which are termed myofibroblasts after activation, acquiring contractile, proliferative, migratory and secretory capability. Sphingosine 1-phosphate (S1P) is a bioactive sphingolipid with well-established effects on angiogenesis, carcinogenesis and immunity. Accumulating evidence demonstrates that this metabolite is involved in the profibrotic inflammatory process through the regulation of pleiotropic cell responses, such as vascular permeability, leukocyte infiltration, cell survival, migration, proliferation and HSCs differentiation to myofibroblasts. S1P is synthesized by sphingosine kinases (SphKs) and many of its actions are mediated by S1P specific cell surface receptors (S1P_1-5_), although different intracellular targets of S1P have been identified. Modulation of SphKs/S1P/S1P receptors signaling is known to result in beneficial effects on various *in vivo* and *in vitro* models of liver fibrosis. Thus, a better knowledge of the molecular mechanisms involved in the modulation of the S1P pathway could help to improve liver fibrosis therapy. In this review, we analyze the effects of the S1P axis on the fibrogenic process, and the involvement of a range of inhibitors or approaches targeting enzymes related to S1P in the abrogation of pathological fibrogenesis. All in all, targeting this pathway offers therapeutic potential in the treatment of hepatic fibrosis.

## Introduction

Hepatic fibrosis is a common disorder in almost all chronic hepatic diseases, such as alcoholic and non-alcoholic steatohepatitis, hepatitis and cirrhosis, among others. In addition to their high prevalence, liver diseases linked to the mechanism of fibrogenesis are of particular importance because they present a great tendency to evolve from their more benign forms, where fibrosis appears, toward cirrhosis and finally to hepatic tumors (Friedman, 2008). However, the molecular mechanisms participating in the development of fibrosis and its evolution to cirrhosis and hepatocellular carcinoma (HCC) are not well established. The most recent advances in the knowledge of the mechanisms underlying fibrogenesis include the appearance of new signal pathways, cytokines and the dynamic activation process of hepatic stellate cells (HSCs). The understanding of the cellular and molecular bases of hepatic fibrosis has increased considerably in the last two decades. Despite the progress made in this field, treatments available for this disease are still limited (Zhang et al., 2016).

Sphingosine 1-phosphate (S1P) is a bioactive lipid mediator, formed by the phosphorylation of sphingosine by sphingosine kinases (SphKs) 1 and 2, which participates in the regulation of a variety of biological activities in different cell types (Pitson, 2011). S1P does not accumulate in cells/tissues under normal conditions, because intracellularly generated S1P is exported and binds to specific cell surface S1P receptors (S1P_1-5_) and can act on intracellular targets before being degraded by S1P lyase (S1PL), a key enzyme involved in the terminal breakdown of S1P intro hexadecenal and ethanolamine phosphate in mammalian tissues (Serra and Saba, 2010). S1P nowadays known to mediate an array of basic cellular processes participating in the response of tissues to injury, like contraction, proliferation, migration, survival or cell interaction (Hannun and Obeid, 2008; Rivera and Chun, 2008; Park and Im, 2017). Among these, the potential of S1P to regulate the fibrogenic process in various organ systems, including the liver, been a matter of interest (Pyne et al., 2013; Schwalm et al., 2013). Although in the last years antifibrotic activity has been revealed for many compounds *in vitro* and in animal models, and different trials have described a high potential of emerging drugs to improve liver fibrosis of diverse etiologies, there are still some target proteins and pathways that remain to be elucidated (Trautwein et al., 2015; Bansal et al., 2016). This review analyzes the current knowledge on the role of S1P signaling pathway as an essential factor involved in the molecular mechanisms of hepatic fibrogenesis, and also focuses on drugs targeting the SphKs/S1P/S1P receptors axis, which constitute a potential therapy in the treatment of liver fibrosis.

## Mechanisms Involved in the Pathogenesis of Liver Fibrosis

Liver fibrosis is a reversible wound-healing response resulting from a wide variety of etiologies. Different cellular and molecular events that lead to an accumulation of collagen and extracellular matrix (ECM) protein in the space of Disse are involved, generating an cirrhotic process with high morbidity and mortality (Pinzani and Rombouts, 2004; Novo et al., 2014; Pellicoro et al., 2014). Alterations in the wound healing mechanism may disrupt the normal architecture of the hepatic tissue causing its dysfunction and failing (Rosenbloom et al., 2010; Hams et al., 2015). The insult to different organ specific cells can lead to the release of damage-associated molecular patterns (DAMPs), along with proinflammatory and profibrotic factors (Hams et al., 2015). Inflammation, endoplasmic reticulum (ER) stress and other pathways related to inflammation such as autophagy or apoptosis are included as fundamental disease-regulators (San-Miguel et al., 2015). Oxidative stress also contributes to fibrogenic disorders and to the overexpression of genes involved in scar formation and inflammation (Novo and Parola, 2008).

At early stages of the fibrogenic process, liver sinusoidal endothelial cells, platelets, soluble mediators, transforming growth factor-β (TGF-β), and platelet-derived growth factor (PDGF), among others, induce initiate repair and regeneration through wound healing responses (Krenkel and Tacke, 2017). The activation of HSCs, the main fibrogenic cell type in the liver, is the result of interactions with hepatocytes, macrophages, endothelial cells, cholangiocytes and natural killer cells. This activation and differentiation into myofibroblast leads to the deposition of ECM proteins which participate in the appearance of portal hypertension, progression to liver cirrhosis and cancer (Ogawa et al., 2012). Besides, intracellular pathways such as autophagy, have also been implicated in the activation of HSCs by the supply of energy substrates through the hydrolysis of retinyl esters and the generation of fatty acids (Hernández-Gea et al., 2012). Interestingly, resolution of fibrosis may take place at the same time as senescence, inactivation or apoptosis of activated HSCs (Lee et al., 2015). Matrix can be degraded by a variety of enzymes, but primarily by metalloproteinases (MMPs), that are susceptible to inhibition by tissue inhibitors of metalloproteinases (TIMPs) (Ordoñez et al., 2014). In the healthy liver, ECM is degraded and thus does not accumulate to cause fibrosis (Huang et al., 2017); however, when the TIMPs-MMPs balance is disturbed by hepatic damage, ECM deposition and development of fibrosis increase (Iredale et al., 2013). On the other hand, many studies indicate that if the injury is removed liver fibrosis is reversible; in fact, activated HSCs, hepatocytes, endothelial and immune cells cooperate in the establishment and resolution of liver fibrosis (Campana and Iridale, 2017). Moreover, regression of cirrhosis has been observed in some cases (D’Ambrosio et al., 2012; Marcellin et al., 2013; Pellicoro et al., 2014). Therefore, reversibility is a requirement for the discovery of new targets and the development of customized multi-drug regimens in anti-fibrotic therapy (Friedman and Bansal, 2006).

As a consequence of the large number of biological processes participating in the development of liver fibrosis, a diversity of antifibrotic agents has been tested. Potential approaches to treat fibrosis and promote the resolution of this process are being extensively studied. Among potentially useful strategies, oxidative stress, activation of the farnesoid X receptor, inhibitors of hedgehog signaling, combined peroxisome proliferator-activated receptors (PPAR)-α/δ agonists, improvement of insulin signaling, or manipulation of gut microbiota, among others, have been investigated (Mehal and Schuppan, 2015). Preliminary human studies have found that antioxidants are able to reduce liver inflammation and disease severity, suggesting its usefulness as adjuvant agents in antifibrotic therapy (Czaja, 2014). *N*-acetylcysteine, beyond its antioxidant capacity, exerts antifibrotic effects in CCl_4_-induced liver fibrosis (Morsy et al., 2012; Demiroren et al., 2014), diethylnitrosamine (DEN)-induced fibrogenesis (Mazo et al., 2013), and secondary biliary cirrhosis (Vercelino et al., 2010), by modulating HSCs activation and down-regulating increased expression of profibrogenic genes that contribute to the accumulation of matrix proteins. The combination of *N*-acetylcysteine with metformin reduced hepatic fibrosis in patients with non-alcoholic steatohepatitis (De Oliveira et al., 2008). L-carnitine and genistein (Demiroren et al., 2014) or curcumin and α-lipoic acid (Morsy et al., 2012) induced significant protective effects in CCl_4_-induced fibrosis. Vitamin E is also reported to prevent hepatic fibrosis in animal models and patients with acute and chronic liver disease (Czaja, 2014). Melatonin exerts different effects, such as protection against oxidative stress (Crespo et al., 2010; Das et al., 2017), inhibition of ER stress (Tuñón et al., 2013) and modulation of autophagy response and apoptosis (San-Miguel et al., 2010, 2014, 2015), which contribute to its antifibrotic effect. A main feature of all forms of fibrosis is the altered composition and the increased amount of the ECM. Thus, pharmacological inhibition or genetic deletion of αv integrins attenuates fibrogenesis (Henderson et al., 2013). A humanized antibody (Simtuzumab) that blocks lysyl oxidase (LOXL2) activity and stabilizes fibrillary collagen is at present being evaluated in a large clinical study in patients with liver fibrosis (Gharib et al., 2017). Activation of NADPH oxidases (NOXs) induces HSCs activation (Jiang et al., 2010), and inhibition of NOX1/NOX4 has been shown to suppress fibrogenesis in the bile duct ligation (BDL) and CCl_4_ models (Aoyama et al., 2012; Crosas-Molist and Fabregat, 2015). The immune response, that has multiple interactions with the fibrogenic process, may be also a candidate for therapy (Pellicoro et al., 2014); thus, several strategies to block the TGF-β activity have shown efficacy (Vogt et al., 2011; Rogler et al., 2017), and inhibition of chemokines and their receptors demonstrated antifibrotic effects in rodent models of liver fibrosis (Zaldivar et al., 2010; Seifert et al., 2015; Zubiete-Franco et al., 2017).

Despite what is reported, there is still a need for effective clinical therapies and antifibrotic strategies able to prevent, halt or reverse hepatic fibrosis are required (Campana and Iridale, 2017). In the last years different studies have shown the importance of the sphingolipid pathway in the regulation of fibrosis, and how the beneficial effect of different antifibrotic molecules could be related with the inhibition of the SphKs/S1P/S1P receptors pathway.

## Sphingosine 1-Phosphate: A Signaling and Regulatory Molecule

**FIGURE 1:**
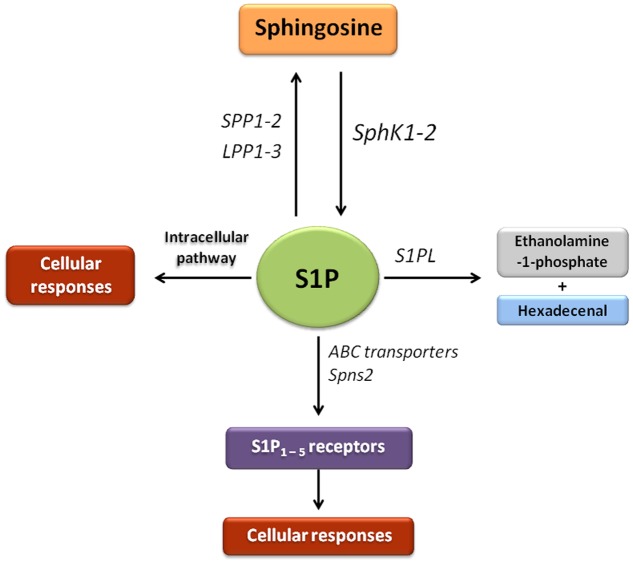
Sphingosine-1-phosphate signaling pathway. In this scheme the enzymes that participate in the synthesis, transport and degradation of S1P are represented. The bioactive molecule S1P can be generated through the phosphorylation of sphingosine by sphingosine kinases (SphK1 and SphK2). S1P is transported across the plasma membrane by ABC transporters and spinsters homolog 2 (Spns2). Most of characterized actions of S1P are mediated through binding to a family of five G protein-coupled receptors referred to as S1P receptor types 1–5 (S1P_1-5_). S1P can be dephosphorylated by the action of two S1P-specific phosphatases (SPP1–2) and three lipid phosphate phosphatases (LPP1-3). The generated sphingosine can be reutilized by SphKs to produce S1P or be available for the synthesis of complex sphingolipids. In addition, irreversible clearance of S1P to ethanolamine-1-phosphate and hexadecenal occurs by the action of S1P lyase (S1PL).

S1P is a potent bioactive lipid mediator synthetised from the substrate sphingosine by SphK1, mainly localized in the cytosol, and SphK2, present in various organelles depending on cell type (Maceyka et al., 2012; Kunkel et al., 2013). SphK1 is the predominant isoform of the enzyme in many cells, and catalyzes the formation of S1P, that exerts a variety of activities including the regulation of a variety of cellular processes important for health and disease (Hait et al., 2009). S1P levels are tightly controlled by sphingosine levels, SphKs, and the enzymes that degrade S1P, which include S1PL, two S1P-specific phosphatases (SPP1-2) and three lipid phosphate phosphatases (LPP1-3) (Maceyka et al., 2012) (**Figure 1**). S1P participates in a range of signaling pathways started by a variety of cytokines, growth factors, hormones and their receptors, such as TGF-β, PDGF, epidermal growth factor (EGF), vascular endothelial growth factor (VEGF), insulin like growth factor 1 (IGF-1), toll-like receptors (TLRs), tumor necrosis factor-α (TNF-α), and protease-activated receptor 1 (PAR-1). S1P functions both as an extracellular and intracellular messenger (Zhang et al., 2013), which exerts different biological functions depending on its site of generation and the SphK implicated (Schwalm et al., 2013). Understanding of the wide range of actions of S1P has been facilitated by the identification of a family of S1P receptors, together with the most recently discovery of intracellular targets (Spiegel and Milstien, 2000; Xia and Wadham, 2011; Adada et al., 2015; Pyne et al., 2016). To activate its receptors, S1P has to be transported across the plasma membrane. ATP-binding cassette (ABC) transporters have been identified as transporters of S1P in different cell lines, such as ABC subfamily C member 1 (ABCC1) in mast cells (Mitra et al., 2006), ABC subfamily A member 1 (ABCA1) in astrocytes (Sato et al., 2007), or ABCC1 and ABC subfamily G member 2 (ABCG2) in breast cancer cells (Takabe et al., 2010). In addition, spinster homolog 2 (Spns2) is probably a specific S1P transporter which has been found in different tissues (Hisano et al., 2011; Fukuhara et al., 2012). When S1P is exported from the cell by these transporters, it binds to specific G protein-coupled S1P_1-5_ receptors to regulate various cellular processes through an autocrine and/or paracrine signaling. S1P_1_, S1P_2_, and S1P_3_ receptors are expressed by a large number of tissues, including liver, while S1P_4_ receptor expression is limited to hematopoietic and lymphoid tissue, and S1P_5_ receptor expression to the central nervous system (Sánchez and Hla, 2004) (**Figure 1**). Activation of S1P receptors participates in different S1P functions which lead to a number of cellular responses, including increased ECM formation, proliferation, stimulation of adherents junctions, inhibition of angiogenesis and apoptosis, or immunity and lymphocyte trafficking (Spiegel and Milstien, 2003; Kee et al., 2005; Brunati et al., 2008).

SphKs are activated by numerous stimuli, including pro-inflammatory cytokines, chemokines and intercellular adhesion molecules (Rivera et al., 2008). S1P is now considered an important player in the cytokine network, and an increasing number of cytokines and other pro- or anti-inflammatory agonists are known to act, at least in part, through the S1P pathway (Xia and Wadham, 2011). Beyond S1P receptors transactivation linked to stimulation by cytokines and growth factors, S1P ligation to its receptors also transactivates growth factor tyrosine kinase receptors; this mutual functional cross-talk has a role in important biological processes such as growth, differentiation, and motility (Donati et al., 2013). Since S1P receptors couple to multiple α subunits of heterotrimeric G proteins and express differentially in distinct cell types, they activate different downstream targets such as small GTPases Rac and Rho and protein kinases ERK, AKT, and JNK, resulting in diverse biological responses, which sometimes are opposite or overlap (Gräler, 2010).

A large part of research has focused until now on S1P signaling through S1P receptors; however, results from different studies had shown in the last few years the presence of direct intracellular targets and it has been demonstrated that S1P binds an alters the function of different intracellular proteins. Thus, S1P has been shown to have transactivating properties in cell proliferation induced by PDGF (Pyne and Pyne, 2002), to inhibit histone deacetylases HDAC1/2 (Hait et al., 2009), to modulate the activity of β–site amiloid precursor protein (APP) cleaving enzyme-1 (BACE-1) and amiloid-β peptide production (Takasugi et al., 2011), and to induce intracellular effects on the TRAF2/RIP1/NF-κB signaling pathway (Alvarez et al., 2010). Mitochondrial S1P also binds to prohibitin 2 (PHB2), a protein involved in mitochondrial biogenesis and metabolism (Strub et al., 2010). Besides this, S1P activation is known to block functions of its precursor ceramide through an intracellular mechanism (Maceyka et al., 2012). Although S1P is generally an important anti-apoptotic mediator (Hait et al., 2006; Tsai et al., 2014; Xu X.Q. et al., 2016), it has been reported to trigger apoptotic signals from human myofibroblasts by an intracellular effect rather than an activation of plasma membrane S1P receptors (Davaille et al., 2002).

Involvement of S1P signaling has been demonstrated in a wide variety of pathogenic mechanisms. Among others, S1P appears to modulate endothelial function and endothelial responses to injury. Recent data suggest that S1P receptors may be potential targets for treatment of not only disorders of the arterial endothelium but also microvascular dysfunction due to ischemic or inflammatory injury (Sanchez, 2016), having been reported that S1P/S1P_2_ receptor contribute partly to endothelial dysfunction by inhibition of the PI3K/AKT signaling pathway (Liu et al., 2016). S1P receptor modulators constitute a new and promising approach to the treatment of a range of inflammatory disorders including inflammatory bowel diseases (IBD) (Nielsen et al., 2017), and data recently obtained in animal models of IBD suggest a contribution of the SphKs/S1P system and related signaling pathways to the anti-inflammatory effect of protocatechuic acid (Crespo et al., 2017). Modulation of the S1P axis may be also useful for the treatment of insulin resistance and progression of type 2 diabetes (Fayyaz and Kleuser, 2014; Ng et al., 2017). Studies have demonstrate an important role of SphK1 in hepatocellular steatosis and shown that deletion of the enzyme reduces hepatosteatosis in diet-induced obese mice (Chen et al., 2016). Research has also shown that S1P production and protein expression of the S1P_1_ receptor were significantly elevated in fulminant hepatitis of viral origin (Crespo et al., 2016). S1P receptors on oligodendrocytes participate in demyelination processes and resultant axonal degeneration in multiple sclerosis (Halmer et al., 2014), and the efficacy of FTY720 has been show in patients with relapsing-remitting disease (Brinkmann et al., 2010). A critical role of SphKs and S1P in carcinogenesis has also been suggested. Thus, overexpression of SphK1 associates to cell proliferation and migration in triple-negative breast cancer (Li et al., 2016), contributing to cell transformation, angiogenesis and metastatic dissemination, and cancer cell multidrug-resistance (Evangelisti et al., 2016; Hatoum et al., 2017). Moreover, S1P levels and expression of SphK1, SphK2, and S1P receptors increase significantly in experimental hepatocarcinogenesis induced by DEN-treatment in mice (Sánchez et al., 2017), and neutralization of S1P reduces hypoxia, modifies vessel architecture and sensitizes to chemotherapy in prostate cancer (Ader et al., 2015).

## Involvement of S1P Signaling in Liver Fibrosis

Different studies have shown that the S1P system is crucial in the regulation of fibrosis disease in numerous organs. Thus, it has been found that SphK1 and S1P receptors play a role in differentiation of mouse and human lung fibroblasts mediated by TGF-β (Kono et al., 2007), in the collagen production by cardiac fibroblasts (Gellings Lowe et al., 2009), or in the transdifferentiation of skeletal myoblasts into myofibroblasts (Cencetti et al., 2010). It is also known that overexpression of S1PL attenuates TGF-β-induced and S1P-induced differentiation of human lung fibroblasts, and reduces fibrosis markers in bleomycin-induced pulmonary fibrosis (Huang and Natarajan, 2015; Huang et al., 2015). In recent years, the S1P axis is also emerging as an important player in hepatic fibrogenesis; S1P is known to have an important influence on several hallmarks of liver fibrosis development (Li et al., 2009a, 2011), since this biomolecule upregulates the expressions of ECM proteins such as TGF-β, alpha-smooth muscle actin (α-SMA) and collagen I and III.

S1P levels in the human fibrotic liver increase through up-regulation of SphKs, regardless of the etiology of fibrosis, and it has been found that there are significant associations between blood sphingolipid metabolites and the stage of liver fibrosis in chronic liver disease, and that S1P may be a candidate biomarker for the identification of HCC in patients with liver diseases (Grammatikos et al., 2016). Moreover, mRNA levels of the S1P transporter Spns2, but not expression of S1PL or SPP1, are enhanced in human liver with advanced fibrosis, indicating an increased export of S1P (Sato et al., 2016). The sphingolipid pathway has also been shown to play a role in mouse CCl_4_-induced liver fibrosis (González-Fernández et al., 2017). It has been found that TGF-β induces activation of mouse bone marrow-derived mesenchymal stem cells (BMSCs) via SphK1-upregulation (Yang et al., 2012), and that production of collagen α1 is increased by SphK1 in human BMSCs and human hepatogenic profibrotic cells after TGF-β treatment (Xiu et al., 2015). *In vitro* experiments show that S1P participates in HSCs activation and differentiation to myofibroblasts, thus contributing to liver fibrosis (Friedman, 2008; González-Fernández et al., 2017). Moreover, S1P is also known to induce HSCs proliferation and migration (Ikeda et al., 2000; Li et al., 2011; Liu et al., 2011). HSCs may play an important role in portal hypertension, a major complication of liver fibrosis; in this regard, S1P has been reported to enhance contractility of HSCs *in vitro* (Ikeda et al., 2000), and to increase portal vein pressure in rats via S1P_2_ receptor with Rho activation (Kageyama et al., 2012; Ikeda et al., 2014). HSCs are also able to participate in angiogenesis, a pathophysiological process closely associated with liver fibrosis, by secreting proangiogenic cytokines, such as angiopoietin 1 (Ang1) and VEGF. Results from *in vitro* studies and murine models of hepatic fibrosis show that stimulation with S1P induces expression of these angiogenic markers via cell surface receptors S1P_1_ and S1P_3_ (Yang et al., 2013). Another important cytokine in the progression of organ fibrosis is PDGF. This cytokine activates SphK1 in HSCs (Brunati et al., 2008), and stimulates the S1P_1_ receptor activity in several fibroblasts cell lines (Hobson et al., 2001; Rosenfeldt et al., 2001). In addition, PDGFRβ has been related to the stimulation of S1P_1_ receptor in mouse embryonic fibroblasts (Long et al., 2006). However, a complex interplay exists between PDGFR and S1P receptors (Pyne and Pyne, 2002, 2008, 2017), and it has been reported that in embryonic fibroblasts from S1P_2_ receptor-null mice S1P_2_ receptor acts as a negative regulator of both migratory and proliferative responses to PDGF (Goparaju et al., 2005).

Although there are reports that S1P participates in collagen deposition in an S1P receptor-independent manner (Xiu et al., 2015), the contribution of S1P receptors to the pro-fibrogenic effect of S1P was reported *in vitro*, in murine models of liver fibrosis (Schwalm et al., 2013; Takuwa et al., 2013) and in patients with advanced fibrosis (Sato et al., 2016). In fact, S1P induced-migration via S1P_1_ and S1P_3_ receptors was demonstrated in activated HSCs (Brunati et al., 2008), human HSCs line LX-2 (Liu et al., 2011), bone marrow-derived monocyte/macrophage (BMMs) migration in mouse models of cholestatic liver injury (Li et al., 2009a,b; Yang et al., 2013, 2015), human hepatic myofibroblasts (hMFs) (Li et al., 2011), and CCl_4_-induced liver fibrosis (Ikeda et al., 2009; González-Fernández et al., 2017).

Regeneration of the liver sinusoidal vasculature is a requirement for regrowth of non-fibrotic liver and restoration of its metabolic capacity (Ding et al., 2016); thus, development of liver fibrosis may result from alteration of the hepatocyte-endothelium crosstalk in the injured organ (Wynn and Ramalingam, 2012). Besides, S1P plays a role in the regulation of various endothelial functions such as vascular maturation, barrier function and flow signaling (Christoffersen et al., 2011; Galvani et al., 2015). Endothelial S1P_1_ receptor is highly expressed in vascular endothelial cells and drives regenerative remodeling of liver, alleviating fibrosis in mouse chronic injury and cholestasis models (Ding et al., 2016).

Some *in vivo* and *in vitro* studies have found that there is a relationship between the development of fibrosis disease and the activation of other pathways like autophagy (Lee et al., 2015; San-Miguel et al., 2015). Although autophagy triggers divergent and cell-specific effects during chronic liver injury (Mallat et al., 2014), the autophagic process has been implicated in driving HSCs by providing important energy substrates through the hydrolysis of retinyl esters and the generation of fatty acids (Hernández-Gea et al., 2012). Down-regulation of the autophagic markers LC3 or beclin1 augments TGF-β-induced expression of fibronectin and α-SMA in human lung fibroblasts (Patel et al., 2012), and it has been shown that over-expression of S1PL attenuates TGF-β-induced S1P levels, and expression of α-SMA in lung fibroblasts through up-regulation of the autophagic process (Taniguchi et al., 2012). Therefore, S1P-mediated autophagy has been identified as an important pathway of fibrosis disease (Tatler and Jenkins, 2015).

In summary, the involvement of the S1P axis in such a range of pathogenic mechanisms related with the fibrogenic process makes it a desirable drug target, and strategies to reduce S1P signaling could be useful for treatment of patients with liver fibrosis.

## Targeting Sphingolipid Metabolism as a Therapy in Liver Fibrosis

**FIGURE 2:**
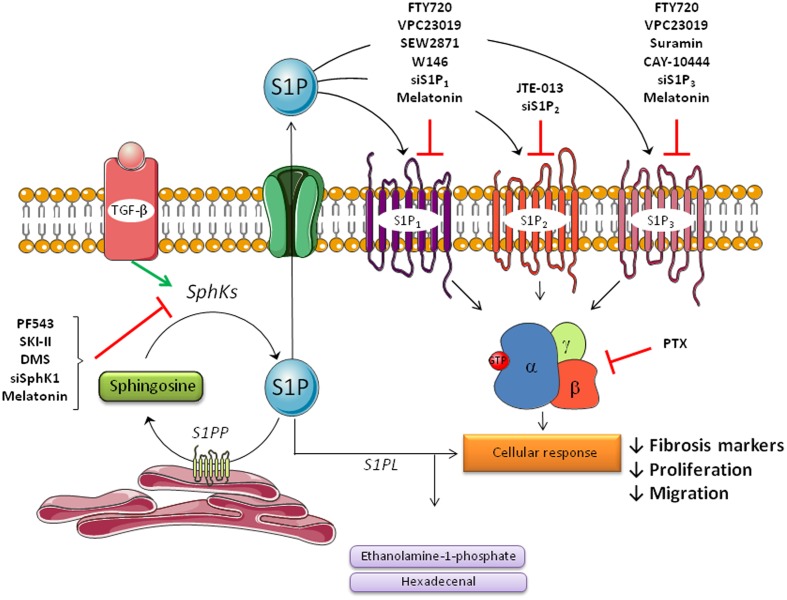
Agents that mediate an antifibrotic action in the liver via regulation of sphingosine-1-phosphate signaling pathway in hepatic stellate cells (HSCs). Hepatic stellate cells play a major role in liver fibrosis by triggering the progression of the disease. During the process of liver fibrosis, quiescent stellate cells are activated by several molecules like TGF-β or PDGF-β to transform in activated cells, which produce extracellular matrix proteins, such as collagen or α-SMA. S1P is involved in these processes primarily via S1P formation by SphK1 and subsequently via S1P receptors (S1P_1_, S1P_2_, and S1P_3_). Several molecules that play an inhibitory action on the sphingolipid pathway are shown. Most of these inhibitors can be used as a therapy in fibrosis disease because of its beneficial effects.

The development of inhibitors of S1P signaling and approaches targeting enzymes involved in the sphingolipid pathway, is a novel area in the search for efficient antifibrotic drugs (Dyckman, 2017; Park and Im, 2017). The remainder of this review will focus on studies in animal models and *in vitro* models of liver diseases which have explored existing drugs or novel therapeutic agents that mediate an antifibrotic action in the liver via regulation of the S1P pathway (summarized in **Table 1** and **Figure 2**). In any case, cautiousness in data interpretation is required considering that compounds and inhibitors are never exclusively acting on one target.

**Table 1:** Overview of sphingolipid targets in liver fibrosis.

Target	Drug/methods	Experimental model	Main findings	Reference
SphK inhibitors	PF543	TGF-β1-activated LX-2 cells	↓ α-SMA	González-Fernández et al., 2017
			↓ Collagen I	
	DMS	HSCs-expressing Dyn2K44A	↓ HSCs migration	Wang Y. et al., 2017
			↓ AKT phosphorylation	
		TGF-β1-activated hMSCs and hHPCs	↓ Collagen I and III	Xiu et al., 2015
		PDGF-activated HSCs	↓ α-SMA	Brunati et al., 2008
			↓ PDGF proliferative effect	
			↓ PDGF-induced cell migration	
Non-selective SphK inhibitors	SKI-II	BDL/CCl_4_-induced liver fibrosis in mice	↓ α-SMA	Yang et al., 2013
			↓ Collagen I and III	
			↓ TIMP1	
			↓ TGF-β1	
			↓ Ang1, CD31, VCAM-1, vWF	
		TGF-β1-activated LX-2 cells	↓ α-SMA	Ge et al., 2016
			↓ Collagen I	
	SKI-II+DMS	TGF-β1-differentiated BMSCs from CCl_4_-induced liver fibrosis in mice	↓ α-SMA	Yang et al., 2012
			↓ Collagen I and III	
			↓ Differentiation to myofibroblasts	
S1P_1_ and S1P_3_ receptors agonist/ functional antagonist	FTY720	PDGF-activated HSCs	↓ α-SMA	Brunati et al., 2008
			↓ S1P proliferative and mitogenic effect	
			↓ PDGFR-β tyrosine phosphorilation	
			↓ PDGF-induced cell migration	
		CCl_4_-induced liver fibrosis in mice	↓ α-SMA	Kong et al., 2014
			↓ Procollagen I and III	
			↓ TGF-β1	
			↓ S1P-dependent BMSCs migration	
		CCl_4_/methionine-choline-deficient diet-induced liver fibrosis in mice	↓ α-SMA	King et al., 2017
			↓ Collagen I	
			↓ Hydroxyproline	
			↓ S1P-dependent cell migration	
		HSCs-expressing Dyn2K44A	↓ Dyn2K44A-induced HSCs migration	Wang Y. et al., 2017
			↓ AKT phosphorylation	
S1P_1_ and S1P_3_ receptors antagonist	VPC23019	PDGF-activated HSCs	↓ α-SMA	Brunati et al., 2008
			↓ PDGFR-β	
			↓ S1P proliferative and mitogenic effect	
			↓ PDGF proliferative effect	
			↓ PDGF-BB mitogenic effect	
			↓ PDGF-induced cell migration	
		BDL/CCl_4_-induced liver fibrosis in mice	↓ α-SMA	Yang et al., 2013
			↓ Collagen I and III	
			↓ TIMP1	
			↓ TGF-β1	
			↓ Ang1, CD31, VCAM-1, *v*NF	
		Primary mouse HSCs	↓ S1P-induced Ang1 expression	Yang et al., 2013
		TGF-β1-differentiated BMSCs from CCl_4_-induced liver fibrosis in mice	↓ α-SMA	Yang et al., 2012
			↓ Collagen I and III	
			↓ BMSC differentiation to myofibroblasts	
S1P_1_ receptor agonist/ functional antagonist	SEW2871	BDL/CCl_4_-induced liver fibrosis in mice	↓ α-SMA	Ding et al., 2016
			↓ Collagen I	
			↓ Hydroxyproline	
			↓ Fibrin β-chain	
			↓ Hepatic parenchymal damage after BDL	
S1P_1_ receptor antagonist	W146	LX-2 cells	↓ α-SMA	Liu et al., 2011
			↓ Procollagen I and III	
			↓ Hydroxyproline content	
			↓ S1P-induced LX-2 cells migration and fibrogenic activation	
		CCl_4_/methionine-choline-deficient diet-induced liver fibrosis in mice	↓ S1P-dependent cell migration	King et al., 2017
		Primary mouse HSCs	↓ S1P-induced Ang1 expression	Yang et al., 2013
		TGF-β1-differentiated BMSCs from CCl_4_-induced liver fibrosis in mice	↓ α-SMA	Yang et al., 2012
			↓ Collagen I and III	
			↓ BMSCs differentiation to myofibroblasts	
S1P_2_ receptor antagonist	JTE-013	BDL-induced liver injury in mice	↓ S1P-induced activation of ERK1/2 and AKT	Wang R. et al., 2017
			↓ S1P-induced cell proliferation and migration	
			↓ Total bile acid levels in serum and cholestatic liver injury	
			↓ Inflammation and liver fibrosis	
		BDL-induced liver injury in mice	↓ α-SMA	Yang et al., 2015
			↓ α-SMA	
			↓ Procollagen I and III	
			↓ Collagen I and III	
			↓ TGF-β1	
			↓ Hydroxyproline	
			↓ S1P-induced BMMs migration	
			↓ BMMs population	
		BDL-induced liver injury in rodents	↓ Portal vein pressure	Kageyama et al., 2012
		LX-2 cells	↓ α-SMA	Xu W. et al., 2016
			↓ Fibronectin	
			↓ Procollagen I	
S1P_3_ receptor antagonist	Suramin	BDL-induced liver fibrosis in mice	↓ α-SMA	Li et al., 2009a
			↓ Procollagen I and III	
			↓ Collagen I and III	
			↓ Hydroxyproline	
			↓ S1P -induced BM cell migration	
			↓ BM cell homing	
		CCl_4_/BDL-induced liver fibrosis in mice	↓ BMSCs migration	Li et al., 2009b
			↓ S1P-mediated homing of BMSCs	
		Cultured hMFs	↓ S1P-induced migration of hMFs	Li et al., 2011
	CAY-10444	BDL-induced liver injury in mice	↓ α-SMA	Yang et al., 2015
			↓ Procollagen I and III	
			↓ Collagen I and III	
			↓ TGF-β1	
			↓ Hydroxyproline	
			↓ S1P-induced BMMs migration	
			↓ BMMs population	
G protein-coupled receptor signaling inhibitor	PTX	PDGF-activated HSCs	↓ α-SMA	Brunati et al., 2008
			↓ PDGFR-β	
			↓ S1P mitogenic effect	
			↓ PDGF-BB mitogenic effect	
			↓ PDGF proliferative effect	
			↓ PDGF-induced cell migration	
		BDL-induced liver injury in mice	↓ S1P-induced BMMs migration	Yang et al., 2015
SphK1 silencing	SphK1 si-RNA	TGF-β1-differentiated BMSCs from CCl_4_-induced liver fibrosis in mice	↓ α-SMA	Yang et al., 2012
			↓ Collagen I and III	
			↓ BMSCs differentiation to myofibroblasts	
		TGF-β1-activated hMSCs and hHPCs	↓ Collagen I and III	Xiu et al., 2015
		TGF-β1-activated LX-2 cells	↓ α-SMA	Ge et al., 2016
			↓ Collagen I	
S1P_1_ receptor silencing	S1P_1_ receptor si-RNA	Cultured hMFs	↓ S1P-induced migration of hMFs	Li et al., 2011
		Primary mouse HSCs	↓ S1P-induced Ang1 expression	Yang et al., 2013
		LX-2 cells	↓ α-SMA	
			↓ Procollagen I and III	
			↓ Hydroxyproline	
			↓ S1P-induced LX-2 cells migration	
S1P_2_ receptor silencing	S1P_2_ receptor si-RNA	BDL-induced liver injury in mice	↓S1P-induced BMM migration	Yang et al., 2015
			↓ BMMs population	
S1P_3_ receptor silencing	S1P_3_ receptor si-RNA	Cultured hMFs	↓ S1P-induced migration of hMFs	Li et al., 2011
		Primary mouse HSCs	↓ S1P-induced Ang1 expression	Yang et al., 2013
		LX-2 cells	↓ α-SMA	Liu et al., 2011
			↓ Procollagen I and III	
			↓ Hydroxyproline content	
			↓ S1P-induced LX-2 cells migration and fibrogenic activation	
		BDL-induced liver fibrosis in mice	↓ S1P-induced BM cell migration	Li et al., 2009a
		BDL-induced liver injury in mice	↓ S1P-induced BMMs migration	Yang et al., 2015
			↓ BMMs population	
Melatonin receptors agonist	Melatonin	TGF-β1-activated LX-2 cells	↓ α-SMA	González-Fernández et al., 2017
			↓ Collagen I	
			↓ SphK1	
			↓ S1P_1_ and S1P_3_ receptors	

### Sphingosine Kinase Inhibitors

#### PF543

PF543 is a potent and specific inhibitor of SphK1 useful for identifying specific roles of SphK1-driven S1P signaling (Schnute et al., 2012), which has shown an inhibitory action on liver fibrosis through the S1P pathway. Treatment with PF543 results in a decrease of SphK1 protein concentration and it also induces a significant impairment of α-SMA and collagen expression in TGF-β1-activated LX-2, thus ameliorating the differentiation toward fibroblasts (González-Fernández et al., 2017).

#### SKI-II

SKI-II (4-[[4-(4-chlorophenyl)-1,3-thiazolyl]amino]phenol) is a well-characterized non-selective SphK1 inhibitor whose administration has been shown to inhibit the fibrogenic process. Liver injury is significantly decreased after SKI-II administration in BDL or CCl_4_-treated mice, with a reduction of transaminases level, accompanied by a marked decrease in mRNA levels of the fibrotic markers, α-SMA and collagen I (Yang et al., 2013). SKI-II is also reported to attenuate the up-regulation of α-SMA and collagen I expressions in TGF-β1-activated LX-2 cells (Ge et al., 2016), and to block the upregulation of angiogenic markers and collagen deposition in HSCs (Yang et al., 2013).

#### *N,N*-dimethylsphingosine

*N,N*-dimethylsphingosine (DMS) is a SphK1 and SphK2 inhibitor (Jung et al., 2017), that also inhibits other enzymes such as protein kinase C (Igarashi et al., 1989), 3-phosphoinositide-dependent kinase 1 (King et al., 2000), sphingosine-dependent protein kinase (SDK) (Hamaguchi et al., 2003), and ceramide kinase (Sugiura et al., 2002). DMS abolishes AKT phosphorylation in Dyn2K44A cells, and inhibits HSCs migration, thus reducing liver fibrogenesis (Wang R. et al., 2017). Furthermore, a reduction of collagen deposition has been shown in human BMSCs (hMSCs) and in primary human hepatogenic profibrotic cells (hHPCs) after DMS treatment (Xiu et al., 2015). The α-SMA accumulation after PDGF activation was also reduced with DMS administration in HSCs (Brunati et al., 2008). The inhibitor reduced the proliferative effect and the cell migration mediated by PDGF in activated HSCs (Brunati et al., 2008). The combination treatment of SKI-II and DMS reduced collagen and α-SMA accumulation and abrogated the prodifferentiating effect of TGF-β in BMSCs from a mouse model of CCl_4_-induced liver fibrosis (Yang et al., 2012).

### Agonizing/Antagonizing S1P Receptors

#### FTY720

The most widely studied drug that regulates sphingolipid effects on fibrosis is the S1P pathway suppressor FTY720 (fingolimod). *In vivo*, fingolimod is phosphorylated by SphK2; the phosphorylated form is an agonist of S1P which binds to S1P_1_, S1P_3_, S1P_4,_ and S1P_5_ receptors (Billich et al., 2003; Sanna et al., 2006). The binding of FTY720 causes internalization and degradation of these receptors, resulting in their downregulation and making the drug to act as a “functional antagonist” (Xie et al., 2017). Protective effects of FTY720 treatment have been shown in various animal models of fibrosis, with attenuation of inflammatory responses or inhibition of the microvascular endothelial dysfunction (Liu et al., 2013; Ni et al., 2013a,b). FTY720 treatment resulted in a reduction of α-SMA deposition, a marked decrease in PDGF-β tyrosine phosphorylation, and partial blocking of the S1P-mediated mitogenic, migratory and proliferative response of cultured PDGF-activated HSCs both in a S1P receptor-dependent and S1P receptor-independent way (Brunati et al., 2008). This drug also has a beneficial effect on liver fibrosis in the CCl_4_-induced mouse model by suppression of BMSCs migration (Kong et al., 2014). Moreover, using mice models with CCl_4_-induced fibrosis, it has been demonstrated that FTY720 results in an attenuation of liver injury, with a significant decrease of transaminases levels and fibrosis markers such as α-SMA, procollagen, TGF-β1 (Kong et al., 2014), or hydroxiproline content (King et al., 2017). In a recent study it has been demonstrated that FTY720 administration for 2 weeks to mice with non-alcoholic steatohepatitis (NASH) results in a reduction in liver injury, inflammation, and fibrosis (Mauer et al., 2017). Research has also shown that the anti-fibrotic effect of injected hematopoietic stem cell in mice with liver fibrosis was augmented with the addition of FTY720 (King et al., 2017). FTY720 administration also abolished AKT phosphorylation in Dyn2K44A cells, and inhibited HSCs migration, thus abrogating liver fibrosis (Wang R. et al., 2017)

#### VPC23019

VPC23019 is a competitive antagonist of S1P_1_ and S1P_3_ receptors, which plays an inhibitory action on the cellular response mediated by S1P receptors. The inhibition of these receptors triggers a decrease of the proliferative and mitogenic effect of S1P in PDGF-induced HSCs, and an attenuation of cell migration, along with a reduction of α-SMA and PDGFR-β (Brunati et al., 2008), collagen I and III deposition, TIMP1, TGF-β1 and transaminases levels (Yang et al., 2013). Administration of VPC23019 in BDL/CCl_4_-induced liver fibrosis and mouse HSCs, induced an significant inhibition of angiogenesis and attenuated the extent of liver fibrosis (Yang et al., 2013). BMSCs differentiation to myofibroblasts was also inhibited after the antagonist administration in TGF-β1-differentiated BMSCs (Yang et al., 2012).

#### SEW2871 S1P1 Receptor Agonist/Functional Antagonist

SEW2871 is a highly selective S1P_1_ receptor agonist/functional antagonist which does not act on the other S1P receptors. SEW2871 was found to protect several organs from injury, including liver failure due to ischemia and reperfusion in mice (Hofmann et al., 2009; Park et al., 2010). This molecule had a modulating action on both cholestasis and chronic hepatotoxin-mediated injury models, reducing the hepatic parenchymal damage and fibrosis, as evidenced by decreases of α-SMA, collagen I levels, hydroxyproline content, and fibrin-β chain expression (Ding et al., 2016).

#### W146

W146, a S1P_1_ receptor antagonist, reduced α-SMA, procollagen I and III, along with decreased hydroxyproline content in LX-2 cell lines (Liu et al., 2011). Its administration also blocked S1P-induced LX-2 cell activation and abrogated S1P-induced migration in a dose-dependent manner (Liu et al., 2011). The inhibition of cell migration was also reported after W146 treatment in a mouse model of CCl_4_/methionine-choline-deficient diet (King et al., 2017). The administration of the antagonist was reported to inhibit angiogenesis induced by S1P in primary mouse HSCs (Yang et al., 2013), and to alleviate BMSCs differentiation to myofibroblasts (Yang et al., 2012), thus attenuating liver fibrosis.

#### JTE-013

JTE-013 is a S1P_2_ receptor antagonist (Osada et al., 2002), that has been useful for the study of its functions in different cell types. This molecule has been shown to reduce α-SMA, procollagen I and fibronectin in LX-2 cells (Xu W. et al., 2016). It has also been reported that JTE-013 inhibition of S1P_2_ receptor significantly reduces portal vein pressure in a rat model of BDL-induced cirrhosis, what may abrogate liver fibrosis (Kageyama et al., 2012). Activation of ERK1/2 and AKT signaling pathway can activate NF-κB, which induces expression of various inflammatory genes. Both cell proliferation and inflammation are key contributors to promoting fibrosis under cholestasis conditions. Concerning this, JTE-013 abrogates the activation of ERK1/2 and AKT induced by S1P in mice with cholestasis-induced liver injury (Wang Y. et al., 2017), and inhibits BMMs recruitment, attenuating hepatic inflammation and fibrosis in mice with BDL ligation (Yang et al., 2015; Wang Y. et al., 2017). Although it is well known that S1P_2_ receptor regulates the Rho/Rho kinase pathway to inhibit cell migration (Muppidi et al., 2014), several studies have found that S1P_2_ receptor plays important roles in tumor growth and progression (Ponnusamy et al., 2012; Orr Gandy et al., 2013), indicating that also favors cell migration (Li et al., 2015). In this regard, S1P_2_ receptor inhibitors might play an important role in fibrosis, and it has been shown that JTE-013 prevents EGF-induced cellular invasion (Orr Gandy et al., 2013).

#### Suramin

Suramin is a S1P_3_ receptor antagonist which may be used against fibrosis disease. The therapeutic importance of suramin has been tested *in vitro* and *in vivo* in BMSCs of CCl_4_ and BDL-induced mice fibrosis, respectively. In both cases, it was shown an inhibition of BMSCs migration and homing, thus mediating liver fibrogenesis (Li et al., 2009b). Suramin also reduced α-SMA and collagen deposition, along with a decreased level of hydroxyproline, thus ameliorating hepatic fibrosis induced by BDL. The bone marrow (BM) cell migration and homing were also inhibited. However, the drug did not affect the extent of inflammation and necrosis in the liver (Li et al., 2009a). It has been also reported that this inhibitor enhances S1P-induced migration in hMFs (Li et al., 2011).

#### Other Inhibitors

KRP203 is a structural FTY720 analog with has a greater selectivity for binding to S1P_1_ versus S1P_3_ and S1P_2_ receptors (Khattar et al., 2013). This molecule is phosphorylated by SphK2 to yield the active metabolites KRP203-P, which works as functional antagonist for S1P receptors. This modulator protects mice from Con A-induced liver injury (Kaneko et al., 2006). Antagonism of S1P_3_ receptor through administration of CAY-10444 attenuates liver fibrosis by inhibiting BMMs migration and reducing α-SMA, procollagen and collagen I and III, TGF-β1 and hydroxyproline content in mice after BDL (Yang et al., 2015). Following treatment of LX-2 with VPC24191, a specific S1P_1/3_ receptor agonist, a pronounced increase in α-SMA has been reported (Al Fadel et al., 2016). Pertussis toxin (PTX), a G protein-coupled receptor signaling inhibitor that blocks S1P signaling, reduced PDGF-β and α-SMA concentration, along with an inhibitory action of proliferative and mitogenic effect in HSCs (Brunati et al., 2008). The administration of the drug also inhibited BMMs migration induced by S1P after BDL in mice (Yang et al., 2015).

#### SphK1/S1P Pathway Silencing

SphK1 silencing has been shown to reduce α-SMA, collagen I and III deposition in BMSCs, abrogating the prodifferentiating effect of TGF-β1 (Yang et al., 2012). Moreover, SphK1 siRNA impairs collagen I and III levels in TGF-β activated human BMSCs and human hepatogenic profibrotic cells, reducing human fibrosis development (Xiu et al., 2015). The antifibrogenic effect of silencing SphK1 was also shown in activated LX-2 cells (Ge et al., 2016). Silencing the expressions of S1P_1_ and S1P_3_ receptors in hMFs resulted in reduced S1P-induced migration (Li et al., 2011), which modulates liver fibrosis. The S1P receptors silencing also alleviates angiogenesis induced by S1P in primary mouse HSCs (Yang et al., 2013), and inhibits LX-2 cells migration, along with reduced α-SMA, procollagen I and III, and hydroxyproline content (Liu et al., 2011). S1P_2_ receptor siRNA has been shown to attenuate BMMs population and migration after liver injury induced by BDL (Yang et al., 2015). In BMSCs from a mouse model of cholestasis-induced liver fibrosis, the administration of S1P_3_ receptor siRNA inhibits S1P-induced cell migration (Li et al., 2009b). The silencing of the same receptor inhibits BMMs population and migration, resulting in reduced liver injury (Yang et al., 2015). A gene related to the improvement of liver disease through S1P signaling pathway modulation, the human antigen R (HuR), has also been studied. It has been reported that HuR mRNA levels increase in activated HSCs isolated from livers of BDL mice, contributing to the profibrogenic action of TGF-β (Woodhoo et al., 2012), and recently has been shown that HuR mediates motility of human BMSCs triggered by S1P in liver fibrosis (Chang et al., 2017). Silencing of HuR results in an inhibition of SphK1 activity, blocking the activation of HSCs from CCl_4_ and BDL-induced fibrosis in mice (Ge et al., 2016).

#### Antioxidant Compounds

There is a narrow relationship between fibrosis disease and antioxidant compounds, because oxidative stress activates SphK1, resulting in increased intracellular levels of S1P (Geoffroy et al., 2004; Wang et al., 2005). Some antioxidant molecules have been used in the treatment of fibrosis in several organs through S1P pathway modulation, like epigallocatechin-3-gallate to inhibit the activation of human buccal fibroblasts (Sar et al., 2015) or curcumin to ameliorate diabetic nephropathy in an animal model of renal fibrosis (Huang et al., 2013). In both cases, the improvement of the fibrosis state was provoked by the inhibition of SphK1/S1P pathway. However, the inhibitory action against liver fibrosis has been only studied with melatonin treatment. Melatonin may play a regulatory effect against fibrosis in various organs and tissues, including the liver (Hu et al., 2016) and abrogates activation of HSCs induced *in vitro* (Shajari et al., 2015). Both in a murine model of CCl_4_-induced liver fibrosis and in a line of human HSCs, the inhibition of SphK1/S1P axis has been recently shown to contribute to the antifibrogenic effects of the indole (González-Fernández et al., 2017).

## Conclusion and Perspectives

Liver fibrosis is a dynamic process that results from a range of liver injuries and whose progression leads to cirrhosis. Accumulating evidence supports that S1P is an important mediator of cell functions, being crucially involved in many cellular processes. *In vivo* and *in vitro* studies evidence that sphingolipids can modulate fibrosis disease. We have here discussed the role played by S1P signaling and its implication in the fibrogenic stage of liver disease processes and summarized the results of research showing how targeting enzymes that generate and metabolize S1P as well as its receptors is potentially useful due to the diverse cellular functions involved in fibrosis. All in all, data reviewed set the stage to further evaluation of compounds which have excellent promise for use as adjuvant therapies in liver fibrosis through targeting and modulating the S1P signaling pathway.

## Author Contributions

MT and JG-G conceived and designed the manuscript. All authors contributed to the writing.

## Conflict of Interest Statement

The authors declare that the research was conducted in the absence of any commercial or financial relationships that could be construed as a potential conflict of interest.
